# Factors associated with the knowledge of obstetric danger signs, and perceptions of the need for obstetric care amongst married young women in northern Nigeria

**DOI:** 10.4102/phcfm.v13i1.2557

**Published:** 2021-03-26

**Authors:** Olugbenga Oguntunde, Jabulani Nyenwa, Farouk Yusuf, Dauda Sulaiman Dauda, Abdulsamad Salihu, Irit Sinai

**Affiliations:** 1 UKAid/ Nigeria MNCH2 Programme, Kano State, Nigeria; 2 Palladium, Abuja, Nigeria; 3 Palladium, London, United Kingdom; 4 Society for Family Health, Abuja, Nigeria; 5 Palladium, Washington, DC, United States of America

**Keywords:** knowledge, obstetric danger sign, perception, adolescent women, northern Nigeria

## Abstract

**Background:**

Married adolescents contribute to poor maternal health indicators in many low-and middle-income countries, where restrictive social norms hinder access to, and utilisation of maternal health services. Addressing these barriers is key to improving health outcomes of young mothers and their children.

**Aim:**

This study assessed married young women’s knowledge of obstetric danger signs and perceptions of the need to attend obstetric services.

**Methods:**

A cross sectional descriptive design, interviewing 1624 randomly selected married young women aged 12–25 years. Data were collected in early 2017 using an interviewer-administered questionnaire on mobile phones, and exported into a statistical software for analysis.

**Results:**

We found low levels of knowledge of danger signs, especially those pertaining to the post-partum period. Respondents’ age, literacy and household wealth were significantly associated with knowledge of danger signs across the continuum of care. Awareness of danger signs during delivery, was strongly associated with perceptions of need for antenatal care (odds ratio[OR]= 2.269; *p* < 0.05), and delivery in a health facility (OR = 1.883; *P* < 0.05). Most respondents believed they must wait for their husband’s approval to attend a health facility when in labour.

**Conclusion:**

Our findings show that the low levels of knowledge of obstetric danger signs, low perceptions of the importance of facility delivery, and the need to obtain husband’s permission before seeking care, are highly contextualised and reflect the socio-cultural and economic circumstances of married young women in northern Nigeria. Interventions must consider these cultural context, and include a strong male-involvement component.

## Introduction

Maternal morbidity and mortality remain a global public-health concern, especially in low-and middle-income countries. Although significant progress has been made, countries in sub-Saharan Africa still bear a substantial burden of maternal morbidity and mortality.^1,2^ Adolescent women aged between 15 and 19 years are particularly at risk.^3,4^

Whilst the proportion of married adolescent girls has declined world-wide,^5,6^ more than 700 million women alive today were married before their 18th birthday,^7^ and some 15 million girls still get married annually who are younger than 18 years.^8^ Contraceptive prevalence amongst married adolescent women in sub-Saharan African countries is lower than amongst older married women, and the region contributes to the highest levels of adolescent childbearing globally.^9^

Overall in Nigeria, about 29% of women in union are aged between 15 and 19 years, but the prevalence of child marriage varies widely from one geopolitical zone to another, with the country’s northwest region having figures as high as 76%, compared to about 10% in the southeast. Similarly, the median age at first marriage amongst women aged 20–49 years in Nigeria is 16.7 and 15.9 years for northeast and northwest regions respectively, whilst maternal mortality amongst young married women in northern Nigeria is significantly higher than amongst older women.^9^

Restrictive social norms in some settings hinder young married women’s access to, and utilisation of, reproductive health and obstetric care.^10,11^ Poor utilisation of maternal and child health services amongst adolescent mothers is associated with adverse maternal and perinatal outcomes, and poorer infant and child health.^12^ Sociocultural and demographic characteristics, as well as community level factors have been shown to contribute to poor service utilisation amongst adolescent mothers,^10,13,14^ exacerbated by adolescents’ poor perceptions of their risk of obstetrics complications.^15^

Recognising that access to and utilisation of obstetric services is key to improved health outcomes of young mothers and their children, several reproductive, maternal, newborn and child health (RMNCH) interventions at the community level have focused on addressing barriers to maternal health service utilisation, including creating awareness of danger signs in pregnancy and childbirth, and improving perceptions of need for services, amongst young married women. The Nigeria Maternal Newborn and Child Health Programme (MNCH2), a UK Aid funded 5-year (2014–2019) health programme, was implemented in six focal states in Northern Nigeria. The programme used an umbrella of demand-generated interventions designed to address socio-cultural barriers to utilisation of RMNCH services in the region. Married adolescent and young women discussed their RMNCH needs in a conducive, free and safe environment (Safe Space Initiative [SSI]) and were mentored by healthcare workers on how to overcome the barriers to accessing RMNCH services, and were further linked to other community groups such as emergency transport schemes (ETS) and traditional birth attendants (TBA). This operations-research study was conducted amongst the young women who participated in this programme, and findings were used to guide further programme implementation.

## Methods

The study was undertaken in two of the six MNCH2 focal states – Kaduna and Katsina, where MNCH2 programme interventions were active. These two states are located in one of the six (northwest region) geopolitical zones of the country.

### Conceptual model

**FIGURE 1:**
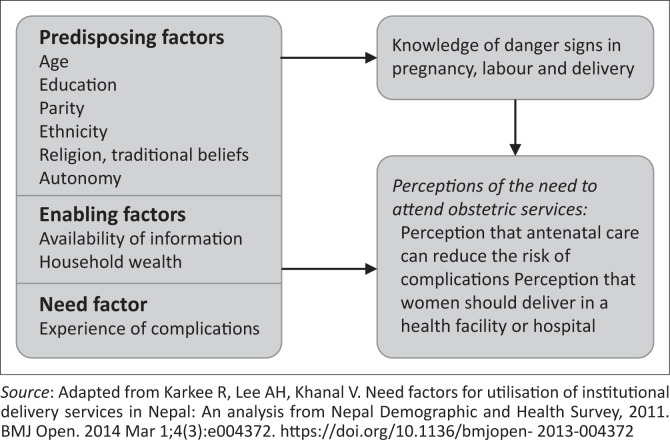
Conceptual framework of factors associated with knowledge of obstetric complications and perceptions of need for services.

We adapted the framework proposed by Karkee et al. (2014)^16^ on determinants of obstetric services utilisation (Figure 1). The framework was based on an earlier seminal work conducted by Andersen et al. (1995),^17^ which outlined a behavioural model that posited need factors that are fundamental to healthcare-seeking behaviour.^16,17^ The Karkee framework further shows predisposing, enabling and need factors as influencing awareness of danger signs, as well as perceptions of women’s need to attend services for obstetric care. Adapting this framework, we assessed young women’s awareness of obstetric danger signs and perceptions of the need to attend obstetric services, which would be an important step towards service utilisation, amongst married young women in communities where these interventions were implemented.

### Study design

We utilised a cross-sectional descriptive study design using a structured interviewer administered questionnaire that assessed young women’s knowledge of obstetric danger signs and their perceptions of healthy behaviours, including attendance at Antenatal care (ANC) and delivering their babies at the health facility in reducing risks of obstetric complications.

### Sample size determination

Using the formula *n*|=(z_1–α_ / δ)^2^ *p*(1 – *p*) for computation of sample size for descriptive studies with *p* = 50%, at 95% confidence interval and precision level of 5%, shows that we can obtain adequate statistical power with a minimum sample size of 384 adolescent women, and increasing to 420 to allow for non-response, as sampling was done at the household, and not the young women level. The full study included elements that required comparisons between four types of communities (beyond the scope of the current manuscript). We therefore planned to interview 1680 (420 × 4) married, young women. The final sample size was 1624 women younger than 25 years who participated in the SSI intervention of the MNCH2-RMNCH programme.

### Sampling technique

To start, we randomly selected one local government area (LGA) from each of the three senatorial districts in each state, for a total of three LGAs. We listed communities in the selected LGAs and removed from the list communities supported by other development partners, to avoid programmatic activities of these partners influencing our results. We then randomly selected two communities from each selected LGA, for a total of 12 study communities in the two states combined.

To identify respondents, we conducted a full-household listing exercise in the selected communities. A total of 9584 households were listed in the two states combined. We randomly selected 2280 households from the list using a random number generator. About two-thirds included at least one married woman younger than 25, and one respondent from each of these households was interviewed. Where we found more than one eligible young woman as members of the same household, one was randomly selected. This resulted in a final sample of 1624 young, married women.

Married adolescent women in Nigeria are considered emancipated adults, and therefore married women aged 12–17 years were interviewed without the need for parent or guardian approval. For the youngest respondents, the interviewers ensured that the adolescents were matured enough to understand the questions before they were included in the study. Interviewer did encounter married women younger than 12 years, but did not invite them to participate in the study.

### Data management

Fieldwork was undertaken in early 2017. We adapted the relevant sections of the women’s questionnaire of the 2013 Nigeria Demographic and Health Survey for the study. The structured questionnaire was administered to study participants by female interviewers, who utilised mobile phones with the SurveyCTO application (Dobility, Cambrige, MA),^33^ to record responses directly. It was undertaken in Hausa, the most commonly used local language in Kaduna and Katsina.

### Data analyses

We exported data into and conducted the analysis using Statistical Package for Social Science (SPSS V 24). We computed descriptive statistics such as means and standard variations for continuous and expressed categorical variables as percentages. We further used cross tabulations and the means to compare characteristics of study respondents in the two states, assessing statistical significance of the differences with χ^2^-square and Analysis of Variance (ANOVA) tests, as appropriate. We present five logistic-regression equations that examined the determinants of knowledge of obstetric danger signs during pregnancy, labour and postpartum period, and perceptions that ANC could reduce risk of obstetric complications should women decide to deliver in a health facility. All the equations included the same independent variables. However, in the perception equation we added the knowledge variables as explanatory variables, in keeping with our framework. This allowed us to examine the association of the explanatory and control variables with perceptions of the need to obtain obstetric care, whilst controlling for their association with the knowledge variables.

We asked respondents about complications in pregnancy, delivery and the postpartum period, and what women can do to minimise the risk. Interviewers did not read the options to respondents. Rather, responses were spontaneous. However, interviewers repeatedly asked, ‘Any other problem?’ until it was clear that respondents mentioned all that they knew. We computed the following dependent variables for multivariate analyses, based on their responses (see Appendix 1 for detailed computation of variables).

### Ethical considerations

Ethical approval for the protocol and study instruments was obtained from the Katsina State Health Research and Ethics Committees (Approval No: MOH/ADM/SUB/1152/1/131) and Kaduna States Health Research and Ethics Committee (Approval number: MOH/ADM/744/VOL1/514) before fieldwork began.

All study procedures complied with international standards for research with human subjects.

Ethical approval for the protocol and study instruments was obtained from the Health Research and Ethics Committees of Kaduna and Katsina states before fieldwork began. In northern Nigeria, the young married woman is often not allowed free social access without her husband’s permission. Therefore, in addition to the precautions we took to protect respondents’ privacy and confidentiality, we put in place special procedures to reduce any physical or emotional risk resulting from study participation. Respondents provided informed consent in private. In some cases, their husbands or other relatives needed to agree to the woman’s participation to reduce risk to the woman. However, the woman was then requested for their consent privately. It was explained to other household members, including the woman’s husband, that she must be interviewed in private and that her responses cannot be shared with them, or else the study results would not be valid. This process allowed us to interview young women who otherwise would not be allowed to participate in the study, whilst ensuring that they were not coerced to participate, and maintaining privacy and confidentiality.

## Results

We interviewed a total of 1624 young women in Kaduna and Katsina states. Respondents in both states were mostly Muslim and married very young (Table 1). Overall, 8.7% of respondents first married when they were 12 years old or younger, and the mean age at first marriage was 15. Parity was also similar in both states. In both states, those respondents with parity >0 had about two children, and most expressed a wish to have many children. More respondents in Kaduna were in polygamous marriages. Literacy levels were low in both states, but lower in Kaduna. About half of respondents in both states worked outside the home.

**TABLE 1:** Respondent profile.

Characteristic	Kaduna *n* = 778	Katsina *n* = 846
Mean age	20.1	19.7
Percent Muslim	94.7***	100.0
Percent in polygamous marriage	36.4***	25.9
Mean age at first marriage	15.4	15.5
Percent who had ever been pregnant	75.1***	80.9
Mean parity	1.4	1.3
Percent pregnant at time of survey	15.3	17.4
Percent literate	27.0***	36.5
Percent work outside the home	47.3**	52.0
Mean ideal number of children	7.9***	9.1
Percent household connected to electricity	55.1	44.7
Mean household wealth index	24.3	23.8

*** and ** denote significance at the *p* < 0.01 and *p* < 0.05 levels, respectively.

We next considered knowledge of obstetric danger signs and perceptions of the need to seek obstetric help. We found that more respondents in Kaduna were aware of danger signs in labour and delivery; they had also experienced significantly more complications as they delivered their own children, but fewer complications in pregnancy (Table 2). Whilst more than three quarters of respondents in both states perceived that antenatal care can reduce the risk of complications, significantly fewer respondents perceived that women should deliver in a health facility or hospital (only about a third of women in Kaduna, and less than a quarter in Katsina). Of interest were the most commonly cited reasons respondents provided for delivering a baby at home: 41.2% of respondents would deliver at home for convenience, 35.1% said delivering at home is safer, and 32.0% said they would receive better care at home. These proportions were similar in both states.

**TABLE 2:** Knowledge and experience of complications of pregnancy, labour and delivery.

Variable	Kaduna *n* = 778	Katsina *n* = 846
**Percent mentioned at least two:**		
Danger signs in pregnancy	50.1	49.9
Danger signs in labour and delivery	64.8***	51.2
Danger signs in first 2 postpartum days	45.9***	37.0
**Percent experienced at least one:**		
Pregnancy complication	32.9***	42.1
Labour complication	34.1***	23.2
First 2 days complication	27.0***	22.3
Percent perceived that antenatal care can reduce the risk of complications	78.4	80.4
Percent perceived that women should deliver in health facility or hospital	32.5***	23.5
**Percent perceived that a woman must have her husband’s permission to go to the health facility if:**		
She is pregnant	89.7	87.6
She thinks that childbirth is starting	86.4***	66.9
She is delivering at home and there is a problem	77.5***	62.8

*** denotes significance at the *p* < 0.01 level.

Although most respondents recognised the importance and utility of antenatal care in reducing complications, almost all said that they would need their husband’s permission to visit a health facility when pregnant. About two-thirds of respondents in Katsina and 86% in Kaduna, believed that when they go into labour, they should wait for their husband’s approval before going to a health facility or hospital, and almost as many would wait for their husband’s approval if they were delivering at home and there was a problem.

Table 3 shows the results of logistic regressions assessing the determinants of the knowledge variables. Having experienced complications in respondents’ own pregnancy, delivery and immediate postpartum period had the most significant positive association with all three knowledge-dependent variables.

**TABLE 3:** Logistic regression of knowledge of danger signs in pregnancy, labour and delivery.

Variable	Odds ratios		
		
	Mentioned at least two danger signs in pregnancy	Mentioned at least two danger signs in delivery	Mentioned at least two danger signs in the first 2 days postpartum
Current age	1.093***	1.212***	1.138***
Age at first marriage	0.924***	0.835***	0.890***
In polygamous marriage	1.283*	1.036	1.040
Parity	0.878**	0.742***	0.714***
Literate	1.085	1.342**	1.101
Works outside the home	0.941	1.162	1.195
Electricity in household	1.288*	1.313**	1.012
Wealth 2nd quartile	1.175	1.224	1.251
Wealth 3rd quartile	1.660***	1.856***	2.168***
Wealth 4th quartile	1.826***	2.040***	2.201***
Experienced at least one complication in pregnancy	10.697***	-	-
Experienced at least one complication in labour	-	2.679***	7.060***
Experienced at least one complication in first 2 days	1.174	1.574***	1.359***
postpartum	0.168***	0.236***	0.149***
Kaduna	-	-	-
Constant	-	-	-
−2 log likelihood	1762.802	1963.446	1890.570

***, ** and * denote significance at the *p* < 0.01, *p* < 0.05 and *p* < 0.1 levels, respectively.

Young women in Kaduna were about one and a half times more likely to know two or more danger signs of both, delivery and the first 2 postpartum days than respondents in Katsina (odds ratios [ORs] of 1.8 and 1.4, respectively). Age was positively associated, but age at first marriage and parity were negatively associated with knowledge. Finally, wealth also had a strong positive association with knowledge. We next examined the determinants of the perceptions that antenatal care can reduce complications and that women should deliver in a health facility or a hospital (Table 4).

**TABLE 4:** Logistic regression of perceptions of healthy behaviours.

Variable	Odds ratios	
	
	Antenatal care can reduce the risk of complications	Women should deliver in health facility or hospital
Current age	1.082***	1.093***
Age at first marriage	0.988	1.108***
In polygamous marriage	1.046	0.714**
Parity	1.109	0.953
Literate	1.731***	2.645***
Works outside the home	0.688***	0.714**
Electricity in household	1.731***	1.543***
Wealth 2nd quartile	1.257	0.955
Wealth 3rd quartile	1.619**	1.092
Wealth 4th quartile	1.419	1.454*
Mentioned at least two pregnancy danger signs	1.343	-
Mentioned at least two delivery danger signs	2.469***	1.883***
Mentioned at least two danger signs first 2 days postpartum	2.095***	1.815***
Experienced at least one complication in pregnancy	1.227	-
Experienced at least one complication in labour	0.869	0.960
Experienced at least one complication in first 2 days postpartum	1.047	1.255
Kaduna	0.632***	1.698***
Constant	0.321**	0.003***
−2 log likelihood	1376.637	1548.025

***, ** and * denote significance at the *p* < 0.01, *p* < 0.05 and *p* < 0.1 levels, respectively.

Our findings show that older respondents and respondents who married younger were significantly more likely, and those in polygamous marriages significantly less likely, to perceive that deliveries should occur in a health facility or hospital. Whilst older women were also more likely to perceive that antenatal care can reduce the risk of complications, marital status and age at first marriage did not have a statistically significant association with this variable. Literacy had a statistically significant positive association and working outside the home a negative association with both perceptions. Wealth was also important – wealthier respondents were more likely to have more positive perceptions of health behaviour. Experiencing complications in their own pregnancies and deliveries did not have a statistically significant association with these perceptions.

Of interest are the findings on the influence of knowledge of obstetric danger signs on the perception of where a woman should deliver her baby. Respondents who recognised at least two danger signs in delivery were almost twice as likely (OR = 1.883) to perceive that women should deliver in a health facility or hospital, compared to those who only recognised one danger sign or none, as were respondents who recognised at least two danger signs in the first postpartum days (OR = 1.815).

## Discussion

Our study confirms that in northern Nigeria, as in many other low- and middle-income countries, knowledge of obstetric signs is poor. Respondents in the two study states, Kaduna and Katsina, were somewhat different. Because of the relative heterogeneity of the population in Kaduna, we expected to find fewer Muslims, fewer women in polygamous marriage, and more literacy amongst women in Kaduna. We observed the opposite, because we only interviewed married women younger than 25 years. Muslim women in northern Nigeria marry younger,^18,19^ and are more likely to become junior wives in polygamous marriages, compared to their Christian neighbours.

Respondents in Kaduna showed better knowledge of danger signs in pregnancy, labour and delivery than respondents in Katsina. These differences were statistically significant. However, when interpreting the differences, we must consider how the variables were defined. We coded the variables as dichotomies, where 1 means the respondent was aware of two or more danger signs, from a list of 10 items for pregnancy, seven for delivery and 13 for the first 2 postpartum days; 0 if they were aware of only one danger sign, or none. For example, when we say that 45.9% of women in Kaduna were aware of two or more danger signs for women in the first 2 postpartum days, we also say that 54.1% knew only one of 13 danger signs, or not even one. Therefore, whilst we can say that knowledge of obstetric danger signs was better in Kaduna, knowledge was very low across the board, consistent with results from other sub-Saharan African countries.^20,21,22,23^

Of interest is the relatively high percentage of respondents who perceived that antenatal care can reduce the risk of complications (more than three quarters), compared to the much lower percentage of respondents who perceived that women should deliver in a health facility or hospital (32.5% and 23.5% in Kaduna and Katsina, respectively). This is consistent with findings reported from other studies, both in Nigeria and in other sub-Saharan African countries.^4,24^ Many other cultural, societal and economic factors enter women’s decision to deliver in a health facility.^10,13,14^ However, since we examine perceptions of the need for hospital care and not the actual behaviour, our findings suggest that attending antenatal care in northern Nigeria may not lead to facility delivery, regardless of these other compounding factors.

In the multivariate analysis of the knowledge variables we found that Kaduna respondents were significantly more knowledgeable. They were more than one and half times more likely than women in Katsina to recognise that women should deliver in a health facility or hospital, but significantly less likely to say that antenatal care can reduce the risk of complications.

Our findings show that predisposing factors influence knowledge of danger signs in pregnancy, delivery and the first 2 days postpartum. We examined age, education, parity and autonomy, and found that older women were significantly more likely to know two or more danger signs in all three knowledge categories. Recognising that these ‘older’ women were still very young (as we only interviewed women younger than 25 years), this makes sense – they have had more opportunity to be exposed to women in their community who experienced complications. Education significantly influenced only knowledge of danger signs in delivery, where better educated women were significantly more likely to know two or more danger signs, as expected.

Both age and education also had a significant positive association with the perceptions that antenatal care can reduce the risk of complications and that women should deliver in a health facility or a hospital. These results were consistent with the literature.^14,23^ It is interesting to note that parity had a statistically significant negative association on all three knowledge variables. Perhaps women with uneventful labour and delivery experiences, tended to be impervious to the possibility of obstetric risks.

We used three variables as proxies for autonomy: age at first marriage, polygamy and working outside the house. We found that women who married at a younger age were significantly less likely to know at least two danger signs in any of the knowledge categories. We can speculate that women who married when they were very young were sheltered in their husband’s home and were less exposed to sources of information about danger signs. Polygamy had a slightly significant positive affect on knowledge of danger signs in pregnancy, but not on knowledge of danger signs in delivery or the first 2 postpartum days. Working outside the home did not appear to influence knowledge of obstetric complications. It is curious, however, that women who worked outside the home were significantly less likely to say that antenatal care can reduce the risk of obstetric complications, and believed that women should deliver in a health facility or hospital.

Of note as we discuss young women’s autonomy is that the majority of respondents believed that they must have their husband’s permission to go to the health facility when they think they are in labour, or if they are delivering at home and there is a complication. Therefore, they did not perceive themselves to be autonomous in this regard. This is consistent with the literature on northern Nigeria, where married women of any age often require their husband’s explicit permission before participating in engagements outside the home.^18^

We used two variables to measure wealth: wealth quartile and availability of electricity in the household. We found that wealth was associated with better knowledge of danger signs, as well as more positive perceptions of the need for obstetric care. This finding is also consistent with the literature.^14,22^

In the analysis of perceptions, we controlled for women having experienced complications in one’s own pregnancy, delivery or the immediate postpartum period. Because having experience with a complication, by definition, means that the woman is aware of the complication, it is not surprising that these variables had the most significant association with all three knowledge categories. These results were consistent with the literature on the effect of complications experienced on knowledge of obstetric danger signs in other African countries.^4,23^

Including the knowledge variables in the perception equations as independent variables, allowed us to assess the association of knowledge of danger signs in pregnancy, delivery and the first 2 postpartum days, with the perceptions of the need to attend obstetric services. We found a very strong positive association. For example, women who mentioned at least two danger signs in delivery were two and a half times more likely to recognise that antenatal care can reduce the risk of complications. The literature shows that inadequate knowledge about obstetric danger signs delays seeking essential obstetric care, with resulting maternal morbidity and mortality.^24,26,27,28^ Young women in our data showed little knowledge of danger signs in pregnancy, delivery and the immediate postpartum period, but those who had better knowledge, also had a better understanding of the need to seek obstetric care, consistent with the literature.

### Limitations of the study

This study has some limitations. First, the cross-sectional descriptive design did not allow for determination of temporal association or causal relationship between the variables. There was also a possibility of recall bias, when we asked respondents about their own experience with complications in pregnancy, delivery and the postpartum period. Another major limitation is that we did not track behaviour (i.e. attending prenatal care; delivering in a health facility). Instead, we used perceptions of the need to attend these services as proxy.

## Conclusion

Overall, our study found low levels of knowledge of obstetric danger signs amongst respondents across the two states with only about half of respondents being able to mention at least two danger signs in pregnancy and childbirth except in Kaduna, where about two-thirds could mention two danger signs during childbirth. Knowledge of danger signs during the postpartum period was much lower with less than half of respondents mentioning at least two danger signs. We also found relatively high understanding of the need for antenatal care, but very low recognition of the need for facility delivery. Whilst low knowledge of obstetric danger signs can be observed in many low- and middle-income countries, these findings should be considered in the context of northern Nigerian culture, where many women marry young and lack autonomy, and where men make household decisions, including those related to obtaining healthcare as revealed by this study. In addition, the respondents’ age, wealth index and literacy are significantly associated with all examined outcomes, including the knowledge of obstetric danger signs and perception of healthy behaviour, including whether attendance at ANC and delivering in health facility could reduce obstetric complications.

Findings of this study suggest that continued efforts to improve married adolescent and young women’s knowledge of obstetric danger signs should emphasise danger signs of complication that may occur throughout the continuum of pregnancy, labour and delivery, as well as the postpartum period. In addition, programmes that focus on improving utilisation of maternal health services such as antenatal care, and delivery at health facilities, must be contextualised to reflect the socio-cultural and economic reality of married adolescent women in northern Nigeria. To maximise the effect of programmes that seek to improve adolescent reproductive health indices, these programmes must incorporate strong male involvement; to increase men’s understanding of the need of young married women for more information, and for autonomy in decision-making when it relates to their health.

## Appendix 1

### Study variables

We asked respondents about complications in pregnancy, delivery and the postpartum period, and what women can do to minimize the risk. Interviewers did not read the options to respondents. Rather, responses were spontaneous. However, interviewers repeatedly asked ‘any other problem?’ until it was clear that respondents mentioned all they knew. We computed the following dependent variables for multivariate analyses, based on their responses:

1. *Knowledge of danger signs in pregnancy.* Serious problems that could occur during pregnancy. We coded responses into the following danger signs: bleeding, severe headache, blurred vision, convulsion, swollen hands or face, high fever, loss of consciousness, difficulty breathing, severe weakness, severe abdominal pain, accelerated or reduced foetal movement, and water breaks without labour. The variable was coded 1 if the respondent listed two or more of the danger signs, 0 if she only mentioned one or none.
2. *Knowledge of danger signs during labour and delivery*. Serious problems that could occur during labour and childbirth. We coded responses into the following danger signs: severe bleeding, severe headache, convulsions, high fever, loss of consciousness, labour lasting more than 12 h, and placenta not delivered soon after birth. The variable was coded 1 if the respondent listed two or more of the danger signs, 0 if she only mentioned one or none.
3. *Knowledge of danger signs to the mother in the first 2 days postpartum.* Serious health problems a mother could experience in the first 2 days after giving birth. We coded responses into the following danger signs: severe bleeding, severe headache, blurred vision, convulsions, swollen hands or face, high fever, vaginal secretions that smell bad, loss of consciousness, difficulty breathing, and severe weakness. This variable was also coded 1 if the respondent listed two or more of the danger signs, 0 if she only mentioned one or none.
4. *Perception that antenatal care can reduce the risk of complications.* Things that a woman can do to reduce the risk of complications in pregnancy and childbirth. This variable is coded 1 if the respondent mentioned antenatal care, 0 otherwise.
5. *Perception that women should deliver in a health facility or hospital.* We asked respondents where, in their opinion, would be the best place to deliver their babies. Our dependent variable was coded 1 if they answered ‘in a health facility or hospital’, 0 otherwise.

We operationalized our framework to include the following explanatory and control variables:

*Age*. This was a continuous variable, ranging 12–24 years. Married adolescent women in Nigeria are considered emancipated adults, and therefore married women aged 12–17 years were interviewed without the need for parent or guardian approval. For the youngest respondents, the interviewers ensured that the adolescents were mature enough to understand the questions before they were included in the study. Interviewer did encounter married women younger then 12 years, but did not invite them to participate in the study.

*Literacy* was used to measure education. This was a dichotomous variable, coded 1 if the respondent could read a simple sentence she was shown, 0 otherwise.

*Age at first marriage.* This was one of three measures of women’s autonomy that we used in the analysis. The assumption was that women who marry very young have less decision-making power.^29^ It is a continuous variable, ranging 12–24, which is the range of age at first marriage of young women in our sample.

*Polygamy*. This variable also measured women’s autonomy, as young women who are junior wives are less autonomous in some settings due to power imbalances.^30^ It was a dichotomous variable, coded 1 if the respondent is in a polygamous marriage, 0 otherwise.

*Parity*. This was a continuous variable, ranging 0–8, which was the range of values obtained from respondents.

*Work.* Our final measure of women’s autonomy was whether or not the respondent was working outside the house, coded 1 if ‘yes’, 0 if ‘no’.

*Household wealth*. We created an index of household goods owned, as a proxy for household wealth.^31^ It did not measure the wealth of the individual respondent, but of the household she was a member of. The index was constructed by collecting information about whether respondent’s household owned, at the time of the survey, each of the following six items: wall clock, mobile phone, fan, radio, television, and refrigerator. For each of the six items we estimated the cost range in the study states at the time of the survey. For example, the cost of the cheapest, used black-and-white television, and the cost of the most expensive new colour television. We then calculated a relative price index for each item, by: (1) dividing the lowest cost of the item by the lowest cost of the cheapest item (wall clock), (2) dividing the highest cost of the item by the highest cost of the cheapest item, and (3) averaging the two. This resulted in a single relative price for each item. Finally, we used the following formula to calculate the value of the wealth index for each respondent.

$${\text{Index}}_{i}=\sum_{i}{\text{OWN}}_{ij}\left(\frac{{\text{RPI}}_{j}}{\sum_{j}{\text{RPI}}_{j}}\right)$$

Where:

OWN_*ij*_ was a dichotomous variable coded 1 if household owned the good *j*, zero otherwise;

RPI_*j*_ was the relative price index of good *j*.

This resulted was an index ranging 0–100, where 100 means the household owned all six items, with a mean of 24.0 and median of 13.4. Because the distribution of this continuous variable was very far from normal, as indicated by the difference between the mean and median and how far they both were from 50, we constructed a series of dummy variables, based on index quartiles, to control for household wealth in the multivariate analysis. The base category was the lowest quartile.

*Electricity*. This was another measure of household wealth, coded 1 if the respondent’s home was connected to electricity, 0 otherwise.

*Experience of complications in labour and delivery.* This variable was coded 1 if the respondent had ever experienced any of the complications listed in the corresponding knowledge variable, 0 otherwise. This was one of two measures of complication experience.

*Experience of complications in the first 2 days postpartum*. This was the second complication experience variable, coded 1 if the respondent had ever experience any of the complications listed in the corresponding knowledge variable, 0 otherwise.

*State*. This variable controlled for the state the respondent lived in, coded 1 if Kaduna, 0 if Katsina. Katsina has a fairly homogenous population. Almost all residents are Muslim, and most belong to the Hausa or Fulani ethnicities, which are culturally similar.^25^ Kaduna is more heterogenous, with a larger proportion of Christians mixed with the Muslims.^9^ This certainly influences behaviour, with Christian women tending to marry later.^32^ Since we selected young married women, most of our Kaduna respondents (95%) were Muslim also. Given this homogeneity, we did not include in the multivariate analysis variables that measure religion and ethnicity. We also did not include a variable on the availability of health information, under the assumption that this is fairly uniform across our study areas.
